# The impact of physical activity on mental health in obese female college students: the role of flow experience in mediation and intrinsic motivation in moderation

**DOI:** 10.3389/fpsyg.2025.1694324

**Published:** 2026-01-13

**Authors:** Zhongsheng Fu, Yunyun Huang

**Affiliations:** Sports and Military Teaching Department, Hainan Vocational University of Science and Technology, Haikou, Hainan, China

**Keywords:** flow experience, intrinsic motivation, mental health, obesity, physical activity

## Abstract

**Background:**

Obesity and mental health are currently among the major global health issues. Obesity not only increases the risk of chronic diseases but also has negative effects on mental health, particularly among female college students. Existing studies primarily focus on the broader college student population, and research specifically addressing obese female college students is insufficient. To address this gap, the present study explores how physical activity influences the mental health of obese female college students, with flow experience as a mediating mechanism and intrinsic motivation as a moderating variable, thus constructing a moderated mediation model.

**Methods:**

A structured survey was conducted with 484 female university students from various institutions in China to gather their self-reported data on physical activity, flow experience, intrinsic motivation, and mental health. Structural equation modeling was employed to test the proposed hypotheses.

**Results:**

The results show that physical activity significantly positively predicts the subjective well-being of obese female college students and significantly negatively predicts depression. Flow experience plays a significant mediating role in this relationship. Additionally, intrinsic motivation significantly moderates the relationships between physical activity and flow experience, as well as between physical activity and depression, nevertheless, it does not notably influence the relationship between physical activity and subjective well-being.

**Conclusion:**

This research contributes to the existing literature on the mental health of obese female college students in the domain of sport psychology and offers a theoretical foundation for designing health promotion programs aimed at this group. The study recommends that future interventions prioritize boosting intrinsic motivation and fostering flow experiences to improve engagement and mental health outcomes for this population.

## 1 Introduction

In recent years, obesity is now recognized as a major global health concern, particularly among young people, with its prevalence continuing to rise, thus drawing widespread attention (World Health Organization, 2000). Obesity is characterized by the excessive or abnormal buildup of fat that can negatively affect health, typically measured by the body mass index (BMI/kg·m^−2^) (Xu et al., 2022). Obesity contributes to a higher risk of cardiovascular diseases, diabetes, and various other chronic health issues (Oppert et al., 2023; Raiman et al., 2023), but also has profound effects on individuals’ mental health (MH) (Öz and Kivrak, 2023). Among female college students, obesity can intensify psychological stress due to factors like academic pressure, societal expectations, and body image concerns, which may contribute to MH challenges such as depression (DEP) and anxiety (Wang et al., 2023). Therefore, it is crucial to explore effective ways to improve the MH of obese female college students, and physical activity (PA), as a non-pharmacological natural treatment, has garnered widespread attention. This study, based on the dual-factor model of MH, assesses the MH levels of obese female college students from both positive and negative psychological dimensions (Wu et al., 2020). Specifically, subjective well-being (SWB) is used to measure positive MH, and DEP is used to assess negative MH. This study offers a fresh perspective on the factors affecting the MH of obese female college students and also provides theoretical support for advancing the overall health development of university students.

Existing research generally supports the positive role of good exercise habits in promoting positive psychology and reducing negative psychological issues such as DEP and anxiety (Garber, 2019; Xu et al., 2022). Research on the relationship between PA and MH primarily focuses on several areas. First, the link between PA and positive psychological outcomes, including how exercise influences self-esteem, self-efficacy (Tikac et al., 2022), psychological resilience (Zhao et al., 2022), and well-being (Wang H. et al., 2022). Second, the connection between PA and negative psychological states, including how exercise influences negative emotions (Qiu et al., 2019), stress (Pinto et al., 2019), interpersonal relationship issues (Du and Liu, 2022), and sleep disorders (Alves et al., 2011). Additionally, research examines how physical activity relates to social support (Liu and Shi, 2023), and PA and cognitive function (Caponnetto et al., 2021).

Despite the increasing amount of research on college students’ MH in recent years, studies specifically focusing on obese female college students remain relatively scarce, and there are certain deficiencies in theoretical perspectives, research content, and cultural contexts. First, existing research primarily concentrates on the MH factors influencing the broader student population, with limited exploration of the health issues of obese female college students within their specific physiological and psychological context. Regarding the relationship between PA and MH, most existing studies have concentrated on the general student population, with little attention to the potential role of flow experience in this relationship and its unique expression in obese populations. Second, many studies overlook the moderating role of intrinsic motivation (IM) in exercise behavior, failing to comprehensively examine the complex interactions between IM, flow experience, and MH, and lacking an in-depth discussion on the mechanisms of MH formation.

In order to address these issues, this study focuses on a specific group—obese female college students. It examines the effects of PA on MH, with a particular emphasis on the mediation of flow experience and the moderation of IM. First, the study focuses on the promotion of MH through flow experience in PA, revealing how PA enhances MH by increasing flow experiences, thus filling the existing gap in this area of research. Second, this study introduces IM as a key variable, investigating how it influences the relationship between flow experience and MH. enriching the research on the interaction between IM and flow experience in sport psychology. Finally, based on the cultural context of China, this study, through empirical analysis of local samples, enhances the cultural adaptability and practical significance of the research, providing theoretical support and practical reference for MH interventions and the advancement of PA participation among obese female university students in China.

## 2 Research hypothesis

### 2.1 Physical activity and mental health

PA encompasses all activities that involve bodily movement and skills, with the primary aim of enhancing physical health and fitness. This involves several elements, such as the kind, frequency, length, and intensity of exercise (Wang K. et al., 2022). Numerous studies provide strong evidence for the connection between PA and MH (Zhang et al., 2023; Zhao et al., 2024). Ai et al. (2021) examined the link between PA and well-being during the COVID-19 pandemic, finding that engaging in suitable PA can greatly enhance overall well-being. Research has indicated that for obese individuals, engaging in exercise significantly boosts positive psychological health, elevating mood and contributing to a greater sense of well-being (Wang H. et al., 2022). For obese female college students, engaging in PA not only aids in weight loss but also improves their physical vitality and enhances positive psychological health (Carraça et al., 2021).

PA is considered an effective method for reducing negative psychological symptoms (Zhu et al., 2020). Participation in PA can alleviate symptoms of DEP and anxiety to some extent, reduce stress, and enhance the ability to cope with psychological pressures (Wang et al., 2004; Zhang and Min, 2022). Additionally, PA stimulates the central nervous system and exerts a positive effect on emotions, demonstrating its potential as an antidepressant (Brand et al., 2020). Zhu et al. (2020) found that appropriate PA could alleviate levels of negative psychology such as anxiety and DEP. However, some studies have noted that excessive PA may lead individuals to feel irritable, increase anxiety and DEP, and cause behaviors such as paranoia and guilt (Mondin et al., 1996). Yet, when the exercise is self-chosen and enjoyable—particularly regarding the type and intensity of exercise—it can effectively reduce DEP and anxiety levels, especially among specific groups such as obese female college students (Baillot et al., 2018). Following the preceding analysis, the study introduces the following hypothesis:

*H1a:* PA among obese female college students significantly positively affects SWB.

*H1b:* PA among obese female college students significantly negatively affects DEP.

### 2.2 Flow experience as a mediator

Flow as described by Csíkszentmihályi (1975), is an optimal psychological state where individuals are completely absorbed in an activity, leading to a range of positive effects. Previous studies have established the beneficial effect of PA on flow experience (Beltrán et al., 2018; Wollseiffen et al., 2016). From a physiological perspective, the occurrence of flow is associated with specific changes in cortical activity, particularly a brief reduction in the function of the inferior frontal cortex, which is related to the increased cognitive performance following PA (Wollseiffen et al., 2016). An extensive analysis performed by Jackman et al. (2021) on adolescent PA and flow revealed that adolescents experience a significant increase in flow during activities such as running and tennis. According to sport psychology, flow is the excitement, engagement, and the sense of fluidity that arises from complete immersion in PA (Wu and Liang, 2011). Liao et al. (2023) found that higher PA demands lead to increased participation in PA and influence flow experiences during exercise among college students. However, the study by Ross and MacIntyre (2020) found that psychological states during exercise, such as personality traits (intelligence and conscientiousness) and emotional regulation, do not significantly affect flow experiences.

Numerous studies have highlighted a robust relationship between flow experience and MH (Ayers-Glassey and Smilek, 2024; Loepthien and Leipold, 2022). Flow as a highly focused and self-actualizing mental state, enables individuals in a flow state to better cope with stress and DEP, demonstrating higher levels of MH (Chen et al., 2019). In addition, flow experience has the potential to boost an individual’s self-efficacy, thereby improving emotional stability and well-being (Lynch and Troy, 2021). Marty-Dugas and Smilek (2020) noted that individuals who tend to engage in deep smartphone experiences generally experience fewer negative emotions like DEP and stress, and are more prone to have positive psychological experiences. By enhancing self-awareness and concentration, flow experience can effectively improve MH and help individuals better adapt to stressors in daily life. Thus, this research suggests that flow experience may act as a mediator in the relationship between PA and MH in obese female college students. Specifically, by enhancing flow experiences in obese female college students, PA can further promote their positive MH and alleviate negative MH outcomes. Building on the previous analysis, the following hypothesis is put forward:

*H2:* Flow experience mediates the effect of PA on MH in obese female college students.

### 2.3 Intrinsic motivation as a moderator

IM refers to the drive to engage in activities based on internal interests, autonomy, and the need for self-actualization, rather than relying on external rewards or pressures (Deci and Ryan, 1985). In the field of sport psychology, IM is considered a key factor influencing an individual’s persistence, engagement, and psychological benefits in PA (Richard et al., 1997). Research has shown that individuals with high levels of IM are more likely to view PA as an enjoyable experience and an opportunity for self-expression, leading to greater enthusiasm and persistence in participation (Teixeira et al., 2012). The link between PA and MH has been broadly established (Zhang et al., 2023; Zhao et al., 2024). Additionally, the flow experience generated during PA (i.e., a highly focused and immersive mental state) has been confirmed to enhance psychological resilience and facilitate emotional regulation (Goddard et al., 2023; Mirvis, 1991). However, the strength of these relationships may vary depending on an individual’s degree of IM. For instance, Kowal and Fortier (2000) discovered that individuals with higher intrinsic motivation were more prone to experiencing greater satisfaction and emotional improvement during PA, leading to more significant improvements in their MH. Furthermore, Jackson and Eklund (2002) highlighted a positive association between IM and flow experience, with individuals possessing high IM being more likely to enter a flow state during exercise, thus enhancing the beneficial impacts of exercise on MH. Recent studies further support the moderating role of IM. Guérin and Fortier (2013) discovered that intrinsic motivation (IM) moderates the effectiveness of PA interventions on the MH of active women, with the high IM group showing significantly greater improvements in mood than the low motivation group. Moreover, the positive effect of flow experience on MH may vary depending on an individual’s level of IM. When individuals participate in activities based on intrinsic interests, the flow state is more likely to translate into long-term psychological adaptation (Mao et al., 2024). Drawing from the theoretical and empirical evidence presented, this study proposes that intrinsic motivation could moderate the intensity of the relationship between PA, flow, and MH by enhancing an individual’s autonomous involvement in PA and deepening the flow experience. Therefore, the following hypotheses are proposed:

*H3a:* IM significantly moderates the link between PA and flow experience in obese female college students.

*H3b:* IM significantly moderates the link between PA and MH in obese female college students.

**Figure 1:**
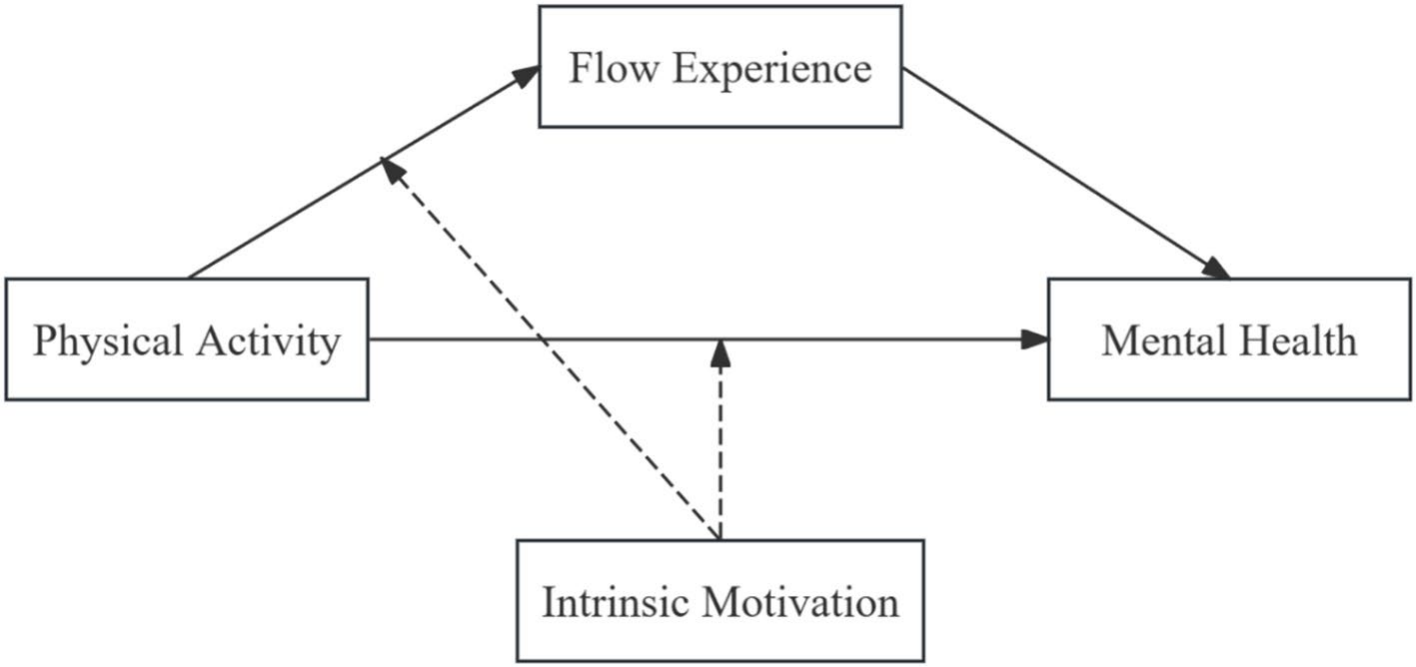
Theoretical model.

The model of this study is presented in Figure 1.

## 3 Materials and methods

### 3.1 Participants

This study was approved by the Ethics Review Committee of Hainan Vocational University of Science and Technology (Approval Number:2025-005), and the research covered multiple universities in China, encompassing the entire process of participant recruitment, data collection, and statistical analysis. All procedures adhered strictly to the international ethical guidelines outlined in the Declaration of Helsinki. A cross-sectional research design was adopted, with convenience sampling used to distribute the questionnaires through the Sojump platform^1^ from March to May, 2025. In order to increase the generalizability and external relevance of the findings, the research team partnered with department heads who provided comprehensive explanations of the survey’s purpose and significance to the students, encouraging their participation. The participants received oral and written information and provided written informed consent before participating in the study.

Following the sample size determination method outlined by Kline (2018), a minimum of 10 respondents is needed for each questionnaire item. Given that the questionnaire in this study had 70 items and accounting for an estimated sample attrition rate of 20%, the final required sample size was calculated to be 840 participants. A total of 900 questionnaires were distributed, and 885 were returned. During further screening, questionnaires from obese female college students were selected based on gender and BMI data, and questionnaires with duplicate IP addresses, those where more than 95% of respondents chose the same answer, those with a response time exceeding three standard deviations, and those missing age information were excluded. In the end, 484 valid questionnaires were retained for analysis (Fu, 2025). Therefore, the final sample for analysis consisted of 484 participants (see Table 1).

**Table 1:** Participant demographics.

Demographic characteristic	Category	Number	Percentage (%)
Age	Under 18 years	148	2.89
	18–22 years	150	93.50
	Over 23 years	186	3.61
Gender	Freshman	342	80.87
	Sophomore	26	8.30
	Junior	69	6.14
	Senior	47	4.69

### 3.2 Measures

#### 3.2.1 Physical activity rating Scale-3 (PARS-3)

The PARS-3, modified by Liang and Liu (1994), was utilized in this study to measure physical activity among college students. The scale consists of three items, which assess the intensity, duration, and frequency of PA. A 5-point Likert scale is used for scoring, from 1 to 5. The PA score = Intensity Score × (Duration Score - 1) × Frequency Score, where higher scores represent higher levels of exercise. Based on the PA score, the activity levels were categorized as low (≤ 19), moderate (20–42), and high (≥43). In this research, the PARS-3 Cronbach’s *α* was 0.726, a KMO of 0.683, and a significant result in Bartlett’s test of sphericity (*p* < 0.001), indicating the scale’s strong structural validity.

#### 3.2.2 Flow experience scale (FES)

The FES employed in this research was created by Jackson et al. (2001). The scale contains 36 items covering nine dimensions: challenge-skill balance, action-awareness fusion, clear goals, sense of control, clear feedback, concentration on the task, time distortion, loss of self-awareness, and autotelic experience. A 5-point Likert scale was used, from 1 to 5. The scale has been thoroughly assessed within Chinese university student populations, proving to be both reliable and valid (Lin, 2023). The scale exhibited a Cronbach’s *α* of 0.955 in this research, with the sub-dimensions showing values of 0.764, 0.806, 0.779, 0.732, 0.781, 0.722, 0.789, 0.819, and 0.851, respectively. Furthermore, the KMO for the entire scale was 0.875, and a significant outcome was found in Bartlett’s test of sphericity (*p* < 0.001), affirming the scale’s solid structural validity.

#### 3.2.3 Exercise motivation scale (MPAM-R)

To assess the level of IM in exercise among obese female college students, this study used the simplified MPAM-R developed by Chen et al. (2013). This simplified scale contains 15 items, a 5-point Likert scale was employed, where higher scores reflected greater levels of IM. Validated within Chinese university student groups, the scale has proven to be both reliable and valid (Zhao et al., 2023). For this research, the MPAM-R’s Cronbach’s *α* was 0.788, and the KMO was 0.846. Furthermore, Bartlett’s test of sphericity was significant (*p* < 0.001), affirming the scale’s strong structural validity.

#### 3.2.4 Depression anxiety stress scale (DASS-21)

The DASS-21, originally created by Lovibond and Lovibond (1995) and revised by Gong (2010), was employed to assess participants’ emotional states from the previous week. The scale contains 21 items, with 7 items each for the DEP, anxiety, and stress subscales. In this study, only the DEP subscale was used. The scale employs a 4-point Likert format, with responses ranging from 1 to 4, and total scores spanning from 7 to 28. A higher score on the scale reflects a higher degree of DEP. This scale has been validated within Chinese university student populations, showing robust reliability and validity (Peng et al., 2025). In this study, the scale’s Cronbach’s *α* was 0.930, with a KMO of 0.935. Bartlett’s test of sphericity was significant (*p* < 0.001), further validating the scale’s strong structural integrity.

#### 3.2.5 Subjective well-being scale (SWBS)

To measure SWB, this study used the SWBS developed by Campbell (1976), which consists of 9 items, including 8 items for overall affective index and 1 item for life satisfaction. A 7-point Likert scale, from 1 to 7, was used, where higher scores indicate a higher level of SWB. Validated in Chinese university student groups, this scale has shown high reliability and validity (Su and He, 2024). With a Cronbach’s α of 0.913 and a KMO of 0.763, Bartlett’s test of sphericity was significant (*p* < 0.001), indicating the SWBS’s robust structural validity.

### 3.3 Statistical analysis

Data analysis was carried out using SPSS 26.0 and AMOS 24.0. Initially, descriptive statistics, correlation analysis, and tests for reliability and validity were performed on the scale data to confirm their fundamental characteristics. Subsequently, Pearson correlation analysis was conducted to explore the relationships between the variables. Mediation (Model 4) and moderation (Model 8) effects were assessed using Hayes (2013) PROCESS macro. For model fit evaluation, 5,000 bootstrap resampling iterations were performed to determine the 95% confidence interval (95% CI). A significance level of α = 0.05 was used, and significance was indicated if the interval did not include zero.

## 4 Results

### 4.1 Collinearity test

In order to ensure model stability and validity, the study carried out an in-depth analysis of multicollinearity among the independent variables. Tolerance and VIF values were calculated with SPSS software to detect any potential multicollinearity problems. The data in Table 2 indicate that all tolerance values were greater than 0.1, and the VIF values ranged from 1.110 to 2.424. A tolerance value greater than 0.1 indicates no severe multicollinearity, and VIF values below 3.3 are considered ideal. This suggests that multicollinearity has an acceptable impact on the study’s results.

**Table 2:** Collinearity diagnostics for the structural model.

Variables	Tolerance	VIF
PA	0.775	1.291
Flow	0.739	1.353
IM	0.927	1.079

### 4.2 Confirmatory factor analysis

In this study, to evaluate the quality of the measurement model, we used confirmatory factor analysis (CFA) to examine whether the factor structure of the questionnaire is consistent with the theoretical framework, ensuring that the questionnaire can reasonably measure the latent variables. According to the results presented in Table 3, the CFA findings reveal that the proposed model exhibits a strong overall fit.

**Table 3:** Model fit indices.

Fit index	Reference value	Final model
CMIN/DF	<5	1.582
RMSEA	<0.05	0.035
GFI	>0.9	0.926
AGFI	>0.9	0.911
CFI	>0.9	0.985
IFI	>0.9	0.985
TLI	>0.9	0.983

### 4.3 Correlation analysis

Table 4 presents the means (M), standard deviations (SD), and correlations for the variables examined in the study. According to Rex B. Kline (2018), skewness values under 3 and kurtosis values under 7 are regarded as acceptable thresholds. PA shows a significant positive correlation with flow experience (*r* = 0.473, *p* < 0.001) and SWB (*r* = 0.563, p < 0.001), and a significant negative correlation with DEP (*r* = −0.534, *p* < 0.01). Flow experience shows a strong positive correlation with SWB (*r* = 0.761, *p* < 0.001) and a significant negative interact with DEP (*r* = −0.614, *p* < 0.001). Therefore, H1a and H1b are supported.

**Table 4:** Correlation analysis of variables.

Variables	M ± SD	SK	Kur	PA	Flow	IM	DEP	SWB
PA	27.140 ± 15.063	0.496	0.868	1				
Flow	131.477 ± 27.400	−1.153	0.319	0.473***	1			
IM	56.300 ± 7.702	−0.989	3.274	0.165***	0.267***	1		
DEP	11.207 ± 4.777	1.509	1.419	−0.534***	−0.614***	−0.267***	1	
SWB	43.746 ± 13.282	−0.853	−0.306	0.563***	0.761***	0.291***	−0.595***	1

*N* = 484, ^***^p < 0.001.

### 4.4 Mediation effect analysis

The mediation effect analysis results are displayed in Table 5. PA significantly positively influences flow experience (*β* = 0.473, *p* < 0.001) and SWB (*β* = 0.262, *p* < 0.001), and significantly inversely influences DEP (*β* = −0.314, *p* < 0.001). Flow experience significantly inversely influences DEP (*β* = −0.465, *p* < 0.001) and significantly positively influences SWB (*β* = 0.638, *p* < 0.001). Therefore, the indirect effects of PA on both DEP and SWB are significant, with indirect effects of −0.220 and 0.302, respectively, and 95% CI of [−0.292, −0.158] and [0.237, 0.368], indicating significant mediation.

**Table 5:** Mediation test of flow experience.

Outcome variable	Predictor variable	*β*	*SE*	*T*	Bootstrap 95% CI		*R* ^2^	*F*
					LLCI	ULCI		
Flow	PA	0.473	0.040	11.788***	0.3942	0.552	0.224	138.946***
DEP	PA	−0.314	0.038	−8.214***	−0.390	−0.239	0.453	199.390***
	Flow	−0.465	0.038	−12.151***	−0.540	−0.390		
SWB	PA	0.262	0.031	8.352***	0.200	0.323	0.633	414.915
	Flow	0.638	0.031	20.339***	0.576	0.699		

^***^Indicates statistical significance at the *p* < 0.001 level.

Following the inclusion of IM as a moderator, Table 6 presents the findings from the moderated mediation analysis. The findings show that PA significantly positively influences flow experience (*β* = 0.441, *p* < 0.001) and SWB (*β* = 0.255, *p* < 0.001), and significantly negatively influences DEP (*β* = −0.323, *p* < 0.001). Flow experience significantly negatively affects DEP (*β* = −0.412, *p* < 0.001) and significantly positively affects SWB (*β* = 0.624, *p* < 0.001). According to the findings in Tables 5, 6, flow experience partially mediates the connection between PA and MH (DEP and SWB) in obese female college students. As a result, H2 is supported.

**Table 6:** Moderating role of IM.

Independent variable	Flow (M)			DEP (Y)			SWB (Y)		
	*β*	*T*	95% CI	*β*	*T*	95% CI	*β*	*T*	95% CI
PA	0.441	11.265***	[0.364, 0.518]	−0.323	−8.604***	[−0.396, −0.249]	0.255	8.152***	[0.193, 0.316]
Flow				−0.412	−10.600***	[−0.488, −0.336]	0.624	19.295***	[0.561, 0.688]
IM	0.178	4.510***	[0.100, 0.255]	−0.088	−2.571*	[−0.155, −0.021]	0.086	3.026**	[0.030, 0.142]
PA × IM	−0.140	−3.910***	[−0.211, −0.070]	0.132	4.259***	[0.071, 0.193]	0.032	1.251	[−0.018, 0.083]
*R* ^2^	0.284			0.482			0.641		
*F*	63.303***			111.371***			213.569***		

*p < 0.05, **p < 0.01, ***p < 0.001.

### 4.5 Moderation effect analysis

**Figure 2:**
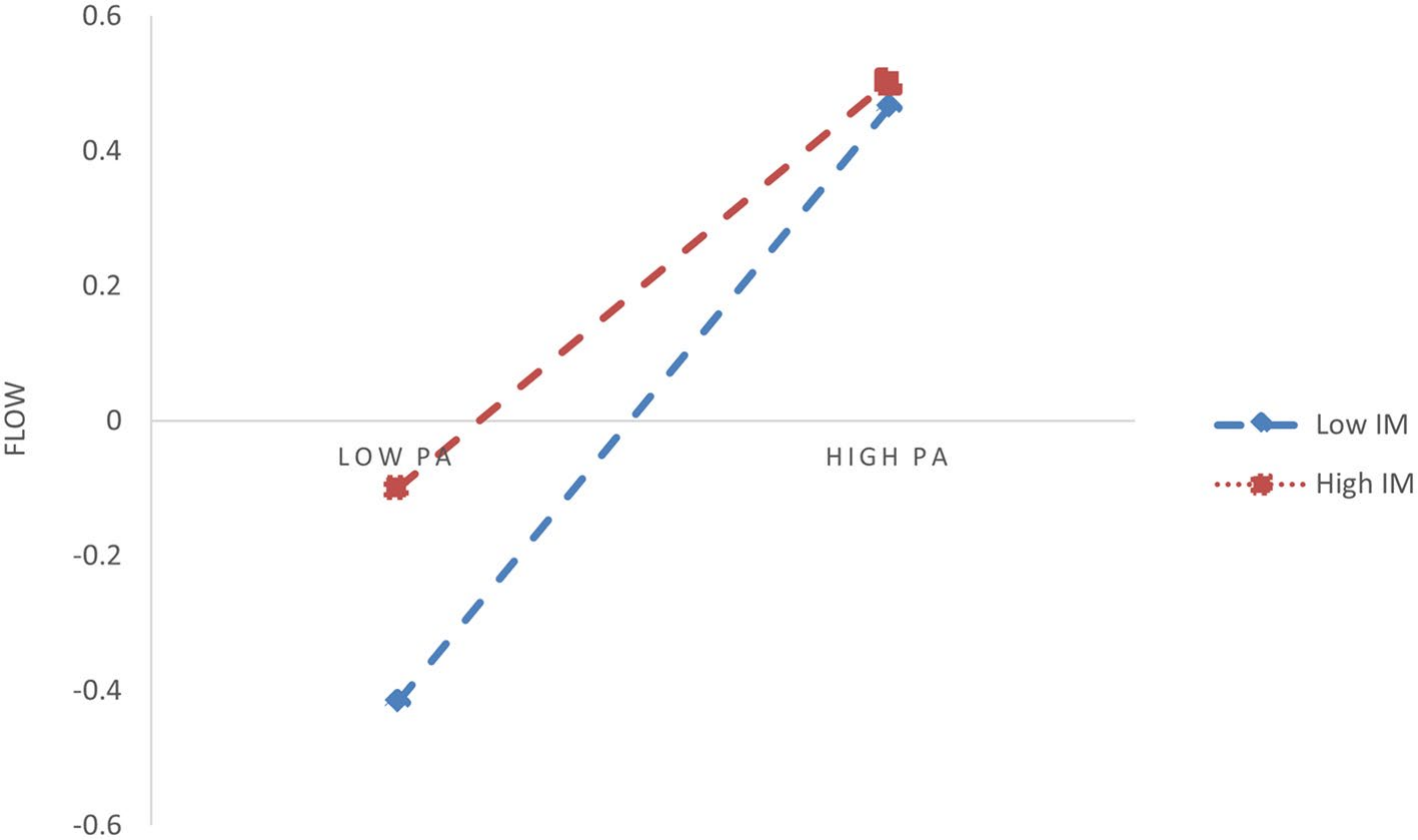
The moderating effect of IM on PA and flow.

**Figure 3:**
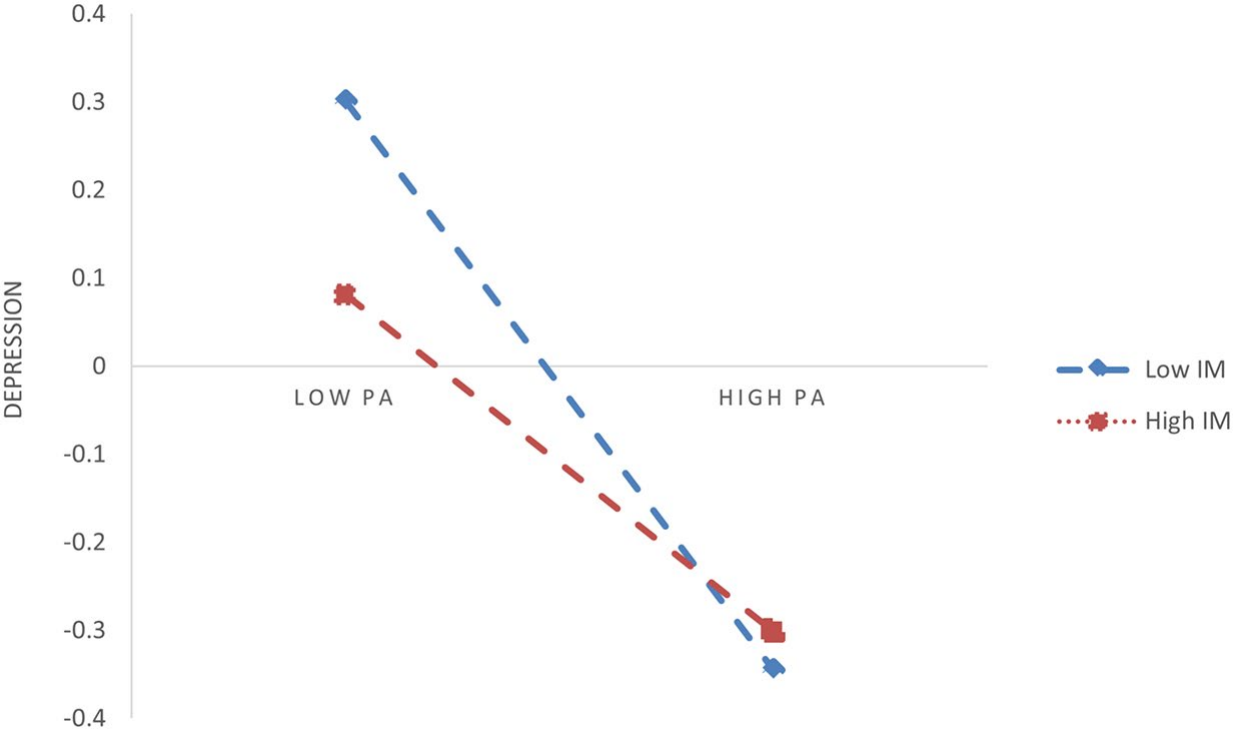
The moderating effect of IM on PA and DEP.

In H3, IM is hypothesized to be a moderating variable that influences the mediation process along certain paths. Based on the standardized scores of IM, participants were categorized into two subgroups: low IM (M - 1SD) and high IM (M + 1SD). As shown in Table 6, the interaction term between PA and IM significantly predicted flow experience. This indicates that IM moderates the relationship between PA and flow experience. Therefore, a simple slope analysis was conducted, and the results are depicted in Figure 2. The results show that, compared to the high IM group (*β_sample_* = 0.301, SE = 0.053, *p* < 0.001), the positive effect of PA on flow experience was further enhanced in the low IM group (*β_sample_* = 0.582, SE = 0.053, *p* < 0.001). Moreover, the relationship between PA and IM was found to strongly influence DEP, indicating that IM plays a key role in shaping the impact of PA on DEP. Simple slope analysis, shown in Figure 3, reveals that compared to the high IM group (*β_sample_* = −0.190, SE = 0.047, *p* < 0.001), the negative effect of PA on DEP was further weakened in the low IM group (*β_sample_* = −0.455, SE = 0.051, *p* < 0.001). However, the bootstrapping results indicate that IM did not significantly moderate the effect of PA on SWB, with a moderation effect index of 0.032, SE = 0.026, 95% CI = [−0.018, 0.083]. Therefore, H3a is supported, and H3b is partially supported.

## 5 Discussion

### 5.1 The connection between physical activity and mental health

Our study revealed that PA is positively correlated with SWB (*r* = 0.563, *p* < 0.001) and negatively correlated with DEP (*r* = −0.534, *p* < 0.001) in obese female college students, supporting H1a and H1b. This supports the findings of Ai et al. (2021), which suggest that appropriate PA can significantly improve overall well-being. Specifically, in obese populations, PA can alleviate DEP and increase SWB by improving physiological conditions, boosting self-confidence, and enhancing emotional (Carraça et al., 2021). Additionally, Zhu et al. (2020) found that appropriate PA can mitigate negative psychological states such as DEP. Regular PA promotes the secretion of endorphins, alleviating depressive feelings and improving MH (Zheng et al., 2024). Furthermore, factors such as enhanced self-efficacy, emotional regulation, and social interaction during exercise may be key mechanisms that increase SWB and reduce DEP (Brand et al., 2020). Overall, the moderate effect sizes indicate that PA plays a meaningful role in improving mental health outcomes. From a practical perspective, these findings suggest that PA interventions for obese female college students should focus on activities that enhance emotional regulation, self-efficacy, and social interaction, such as team sports or yoga. Incorporating flow-inducing elements into PA programs could further amplify the benefits for mental health.

### 5.2 The mediating role of flow experience

Our study first revealed that the mediation of flow experience in obese female college students between PA and MH was statistically significant. Specifically, PA can improve SWB and reduce DEP through flow experience, with indirect effects of −0.220 for DEP and 0.302 for SWB, supporting H2. This discovery emphasizes the crucial role of flow experience in shaping the mental state of those involved in PA. More specifically, PA helps individuals enter a fully immersed psychological state, or flow experience, which enhances emotional and MH (Wu and Liang, 2011). Flow experience, by boosting self-efficacy and emotional regulation, significantly improves SWB and helps individuals more effectively alleviate depressive symptoms (Marty-Dugas and Smilek, 2020). Additionally, the study found that flow experience not only increases focus and enjoyment during exercise but also provides an outlet for emotional release and stress relief, further enhancing MH (Chen et al., 2019). Therefore, flow experience, as one of the ways in which PA influences the mental health of obese female college students, offers a new perspective and practical guidance for optimizing MH intervention strategies. Given the moderate to small effect sizes observed, interventions should prioritize fostering flow experiences during PA, as this can lead to meaningful improvements in both SWB and the reduction of DEP.

### 5.3 Moderating role of intrinsic motivation

The present study indicated that IM significantly moderates the relationship between PA and flow experience, as well as between PA and DEP, but does not moderate the relationship between PA and SWB, thus supporting H3a and partially supporting H3b. Specifically, the effect of PA on flow experience was stronger in the low IM group (*β* = 0.582, *p* < 0.001) compared to the high IM group (*β* = 0.301, *p* < 0.001). Similarly, for DEP, the effect of PA was more pronounced in those with low IM (*β* = −0.455, *p* < 0.001) than in those with high IM (*β* = −0.190, *p* < 0.001). However, IM did not significantly moderate the relationship between PA and SWB (moderation effect index = 0.032, *p* = 0.083). Obese female college students with low IM were more likely to enter a flow state during PA compared to those with high IM, as they tend to rely more on external incentives and the activity itself, facilitating focus and emotional regulation (Bellini et al., 2022). In contrast, those with higher IM may focus more on personal goals, resulting in a weaker flow experience. For the low IM group, PA had a stronger effect on reducing DEP, likely because they lack sufficient self-efficacy and rely more on external support for emotional regulation (Kowal and Fortier, 2000). In contrast, individuals with high IM may manage their emotions more effectively, reducing the impact of PA on DEP. Furthermore, as IM did not significantly moderate the relationship between PA and SWB, this suggests that SWB may be influenced by broader factors beyond motivation, such as physical health and quality of life, which should be considered in future intervention designs.

## 6 Implications and limitations

### 6.1 Implications

This study focuses on obese female college students and explores the dual impact of PA on positive and negative MH, including the roles of flow experience and intrinsic motivation as mediators and moderators in this process. As a result, this study’s findings offer valuable insights for both theory and practice.

From a theoretical perspective, this study expands the theoretical framework on the impact of PA on MH by proposing the dual mechanism of flow experience and IM in the relationship between PA and MH. By revealing that PA influences not only positive psychological states (such as SWB) but also significantly alleviates negative psychological states (such as DEP), this research offers a fresh theoretical viewpoint for both sport psychology and health psychology. Moreover, this study further verifies the moderating role of IM in enhancing flow experience and alleviating DEP during PA. This contributes to the understanding of how motivation factors play a role in MH interventions, enriching the research on the interaction between motivation and flow experience in sport psychology, and advancing the intersection of sport and MH.

From a practical perspective, the findings offer several actionable insights for universities, community health centers, and policymakers. First, to enhance the positive impact of PA on MH, institutions should promote customized PA plans that include not only traditional individual exercises and team sports but also activities like yoga and meditation, which encourage mental health and social interaction. Establishing psychological counseling and health guidance services could help obese female college students build stronger self-efficacy and social support networks. Given the significant negative impact of PA on negative psychological states, universities should strengthen MH education, providing workshops and lectures to educate students on managing stress and anxiety through PA. Additionally, tailored exercise courses, particularly for students with weight management needs, should be developed. These progressive exercise plans would ensure safety while promoting the psychological benefits of PA. Considering the crucial role of flow experience in connecting PA to MH, schools should design physical activities that foster flow. Offering activities that are challenging and provide immediate feedback could help students enter a fully immersed psychological state, enhancing emotional regulation and SWB. Psychological health education and regular emotional regulation training should also be provided to help students manage emotions and enhance their psychological resilience. Lastly, addressing the moderating role of IM, educational and health institutions should create sports programs that aim to enhance IM. For obese female college students, those with low IM may benefit from engaging, interactive physical activities, such as team competitions or customized exercise programs, that help promote emotional regulation and participation. Conversely, for students with high IM, personalized exercise plans focusing on self-regulation and emotional management would further enhance their connection to PA and MH. This research highlights the potential benefits of incorporating both flow experience and intrinsic motivation in PA interventions, ultimately improving mental health outcomes for obese female college students. Given the growing obesity rates, these interventions could be essential in mitigating the long-term psychological and physical health challenges associated with obesity.

### 6.2 Limitations

Although this study has advanced our understanding of the relationship between PA, flow experience, IM, and MH in obese female college students, several limitations exist. Firstly, the cross-sectional design used in this study restricts our ability to infer causal relationships between the variables. While valuable insights have been gained, the design does not allow for a determination of the directionality or long-term effects of PA on MH. Future research employing longitudinal designs could track changes in the same group over time, offering a more robust understanding of how PA, flow experience, IM, and MH evolve in relation to each other. Secondly, the study relies on self-reported data, which may introduce biases, such as those resulting from social desirability or inaccurate recollection. To address these limitations, future research could incorporate objective tools, such as wearable devices, to directly measure PA patterns. Additionally, professional clinical assessments could be used to assess MH status, providing more objective and accurate data. Additionally, this study focused solely on obese female college students, which may not be generalizable to male students or other demographic groups. Gender differences in motivation and emotional regulation may affect PA and MH outcomes, as women may experience PA differently from men. Future research should include male participants to explore potential gender differences. Lastly, the participants in this research are confined to university students from a specific location, which may not fully represent the student populations in other regions or cultural contexts. Future studies should extend the participant base to include students from different cultural contexts and countries, to examine how cultural factors might influence the connection between PA and MH.

## 7 Conclusion

This research investigated the influence of PA on the MH (SWB and DEP) of obese female college students, while also considering the mediating and moderating effects of flow experience and IM. The results show that PA significantly promotes positive psychological health and alleviates negative psychological states, with flow experience playing a significant mediating role in this relationship. Additionally, IM significantly moderates the relationship between PA and flow experience, as well as between PA and DEP, yet, it does not serve as a moderating factor in the relationship between physical activity and subjective well-being. The findings of this study lay the groundwork for developing psychological health interventions targeted at obese female college students, particularly in enhancing IM and flow experience during PA, which can effectively promote emotional regulation, alleviate depressive symptoms, and improve overall MH. Future research can further explore additional potential mediating and moderating variables to deepen the insight into the complex pathways through which physical activity impacts MH.

## Data Availability

The datasets presented in this study can be found in online repositories. The names of the repository/repositories and accession number(s) can be found in the article/supplementary material.

## Footnotes

1
https://www.Sojump.com
